# When Pneumonia Is Not Pneumonia: T-cell Acute Lymphoblastic Leukemia Presenting With Mediastinal Mass, Airway Compression, and Pericardial Effusion in a 4-Year-Old

**DOI:** 10.7759/cureus.113920

**Published:** 2026-08-04

**Authors:** Milan Parajuli, Anu Danai, Srushti Patel, Herve Jacques

**Affiliations:** 1 Pediatrics, Woodhull Medical Center, New York, USA; 2 Pediatrics, Woodhull Medical and Mental Health Center, New York, USA; 3 Pediatrics, Gujarat Medical and Education Research Society, Gandhinagar, IND

**Keywords:** mediastinal mass, pediatric leukemia, pericardial effusion, pneumonia mimic, t-cell acute lymphoblastic leukemia

## Abstract

Pediatric leukemia may initially present in a way that mimics common respiratory infections, particularly when fever, cough, chest pain, and radiographic findings are observed. This overlap may obscure the diagnosis of malignancy, especially when the initial imaging is interpreted as community-acquired pneumonia. T-cell acute lymphoblastic leukemia (T-ALL) is commonly linked with anterior mediastinal masses that may result in airway compression, atelectasis, pleural or pericardial effusions, and cardiopulmonary instability. We describe a previously healthy four-year-old boy who presented with two weeks of fever and dry cough associated with chest pain. He was initially treated with high-dose amoxicillin for presumed pneumonia, but symptoms persisted, prompting the emergency department (ED) visit. On examination, the patient exhibited mild tachycardia and cervical and right axillary lymphadenopathy, while the systemic examinations, including respiratory, cardiac, and abdominal examinations, were unremarkable. Laboratory evaluation showed leukocytosis with circulating blasts, anemia, elevated inflammatory markers, and markedly elevated lactate dehydrogenase. Repeat imaging showed mediastinal enlargement and a potential mass, suggesting a possible leukemia in the presence of blast cells, and necessitated transfer to a higher center within the hospital network that offered subspecialty services. A CT of the chest showed a large anterior mediastinal mass, left mainstem bronchus narrowing, left lower lobe atelectasis, and a large pericardial effusion. Echocardiography demonstrated a moderate to large circumferential pericardial effusion with early signs of tamponade physiology. Flow cytometry of peripheral blood proved T-ALL. The patient was admitted to the pediatric intensive care unit and given tumor lysis prophylaxis, empiric cefepime for fever in the setting of functional neutropenia, corticosteroids for tumor debulking, and pericardiocentesis before initiation of leukemia-directed therapy. Suspicion of malignancy and urgent evaluation should be considered in patients with persistent pneumonia-like symptoms, mediastinal widening, lymphadenopathy, cytopenias or blasts, elevated lactate dehydrogenase, or failure to improve with appropriate antibiotics.

## Introduction

Community-acquired pneumonia is a common pediatric disease presenting as fever and cough confirmed radiologically through a chest X-ray [1]. However, pediatric malignancies such as leukemia and lymphoma can present with similar respiratory symptoms and radiographic findings [2]. Acute leukemia may mimic pneumonia due to pulmonary leukemic infiltration, atelectasis, infiltrates from mediastinal compression, pleural or pericardial effusions, or malignancy-related inflammation [3]. Distinguishing infection from malignancy is of paramount importance, as a delayed diagnosis may predispose to respiratory compromise, cardiovascular instability, and tumor lysis after initiation of therapy [4].

T-cell acute lymphoblastic leukemia (T-ALL) is a subtype of childhood acute lymphoblastic leukemia, which is frequently associated with a high leukocyte count, lymphadenopathy, and anterior mediastinal masses [5]. Large mediastinal masses may cause compression of the tracheobronchial tree or great vessels and are often associated with pleural or pericardial effusions [4]. We present a case of a four-year-old boy who was initially treated for presumed pneumonia and was later diagnosed with T-ALL following the development of persistent symptoms and cervical and axillary lymphadenopathy, with imaging showing mediastinal widening, airway narrowing, and pericardial effusion with early cardiac tamponade.

## Case presentation

A previously healthy four-year-old boy presented to the emergency department (ED) with a dry cough and fever for two weeks, associated with intermittent chest discomfort. He was initially evaluated at urgent care, where a chest X-ray suggested pneumonia. He completed a 10-day course of high-dose amoxicillin, but his symptoms persisted.

He re-presented to the emergency department with persistent fever, fatigue, and decreased appetite. His parents also reported frequent night sweats that had preceded the current illness. He had no weight loss, gastrointestinal symptoms, joint pain, rash, or recent travel. On review of history, he was born at term following an uneventful antenatal and perinatal course, had age-appropriate growth and development, with no known allergies, and was immunized as per schedule. There was no family history of malignancy or hematologic disease.

On presentation, he was afebrile with mild tachycardia, normal blood pressure, and normal oxygen saturation on room air. He was alert, not in distress, and had no pallor. Examination revealed firm, non-tender bilateral cervical lymphadenopathy, with the largest measuring 2x2 cm, and a firm, fixed right axillary lymph node. Respiratory examination was unremarkable, with no respiratory distress and clear breath sounds bilaterally. Cardiovascular examination showed tachycardia with normal heart sounds and no murmurs, rubs, or gallops. There was no hepatosplenomegaly, and the remainder of the examination was unremarkable.

Based on the patient's clinical presentation, the initial differential diagnosis included atypical pneumonia, viral infections, bacterial pneumonia due to a resistant organism, complicated pneumonia (including necrotizing pneumonia or a small empyema not evident on physical examination), and pulmonary tuberculosis, and less likely hyperinflammatory conditions, such as atypical Kawasaki disease. Initial investigations included repeat chest X-ray and general labs, which included complete blood count, serum chemistry, inflammatory markers, and a respiratory pathogen panel.

**Figure 1:**
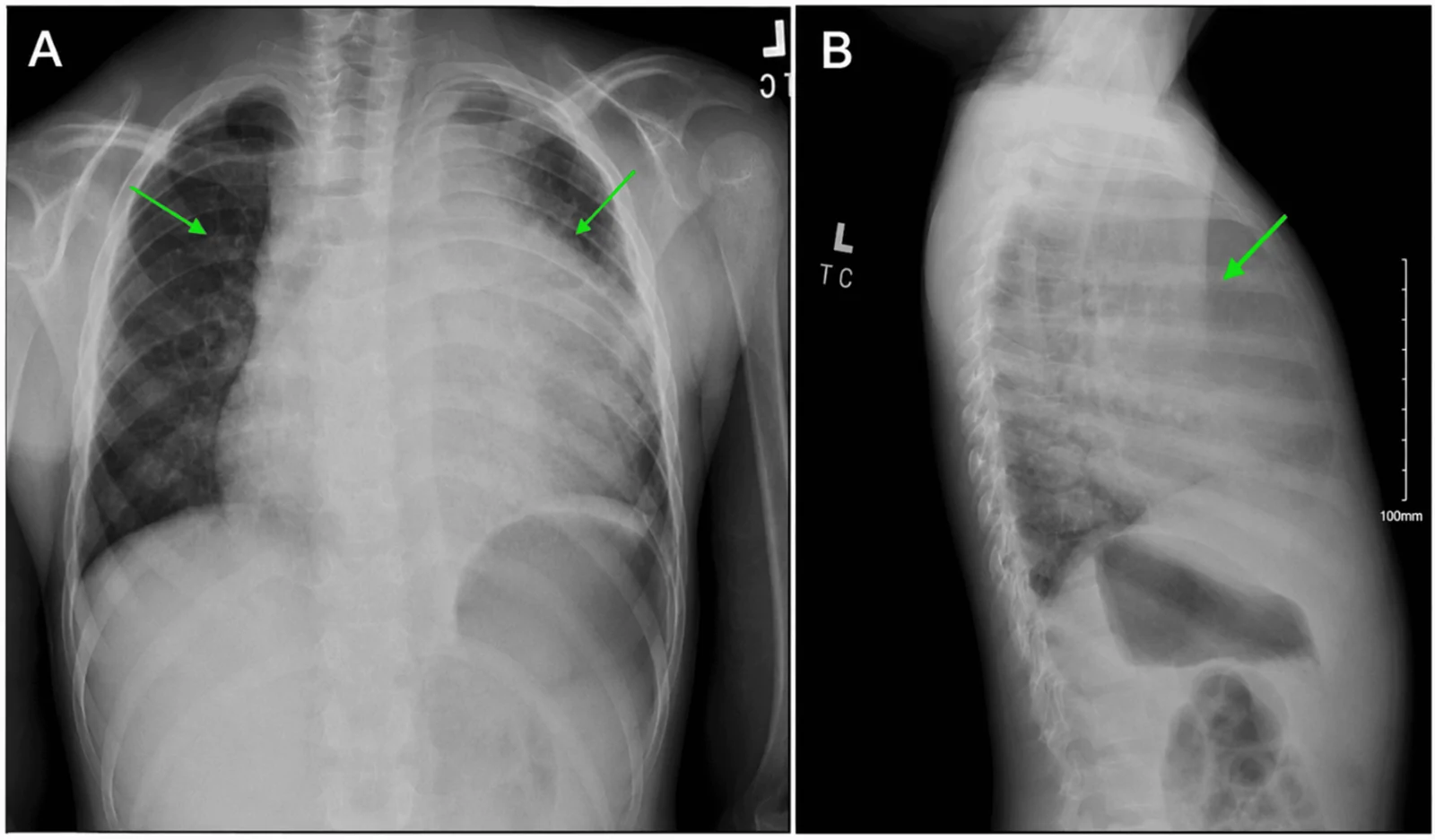
Chest X-ray (A) PA view and (B) lateral view showing mediastinal widening (green arrows) and cardiomegaly.

A repeat chest X-ray demonstrated mediastinal widening and apparent cardiomegaly (Figure 1).

Initial laboratory tests showed hyperleukocytosis with approximately 45% blasts. Hemoglobin showed anemia with a normal platelet count. Inflammatory markers, C-reactive protein, and an erythrocyte sedimentation rate were elevated. Lactate dehydrogenase was elevated, as shown in Table 1. The respiratory pathogen panel was negative for respiratory pathogens, including atypical organisms. Given the combination of circulating blasts and mediastinal widening, an underlying hematologic malignancy became the leading diagnostic consideration. The uric acid, magnesium, calcium, and phosphorus were sent in addition to a basic metabolic panel and were within the normal range and showed no evidence of tumor lysis syndrome. 

**Table 1:** Complete blood count with differentials with inflammatory markers and lactate dehydrogenase levels at presentation to emergency room TLC: total leucocyte count, ANC: absolute neutrophil count, RDW: red-cell distribution width, ESR: erythrocyte sedimentation rate, LDH: lactate dehydrogenase.

Components	Reference Range	At presentation to ER
TLC	5.0-15.5 x 10³/µL	53.95
Neutrophil	22.0-69.0 %	12.9
Lymphocyte	16.0-67.0 %	66.0
Monocyte	4.0-12.0%	20.1
Eosinophil	0.0-4.0%	0.1
ANC	Above 1500/mm³	6960
Hemoglobin	10.2-13.7 g/dl	8.7
Red Blood cell count	3.90-5.00 x 10³/µL	2.96
Mean Corpuscular Volume	75.0-87.0 fL	88.2
Mean Corpuscular Hemoglobin Concentration	32.0-34.7 g/dL	33.3
Mean Corpuscular Hemoglobin	23.7-28.3 pg	29.4
RDW	12.5-14.9%	15.9
Platelet	150-400 x 10³/µL	256
C-Reactive Protein	0-5 mg/L	96.7
ESR	0-10 mm/hr	117
LDH	100-210 U/L	916

**Figure 2:**
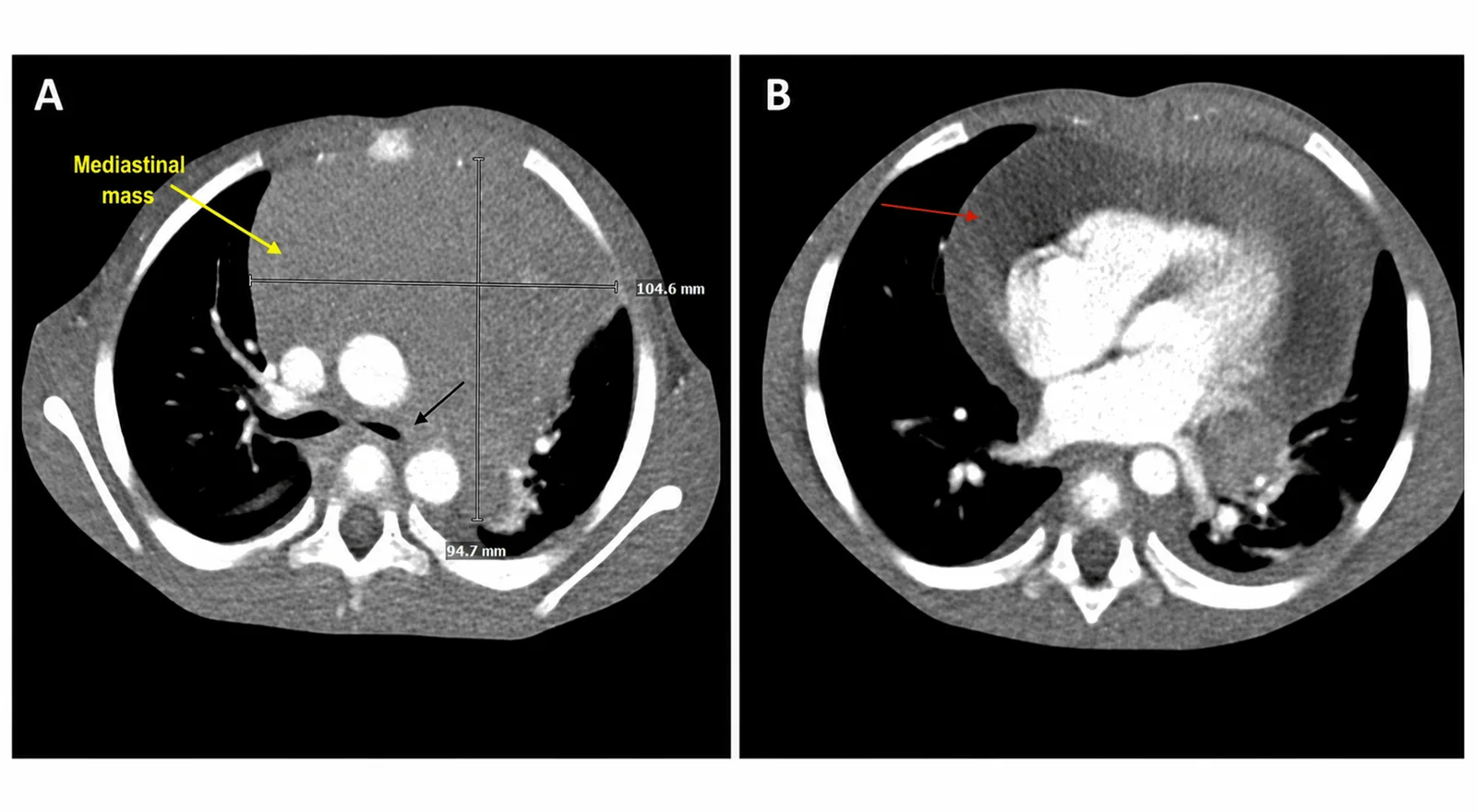
Chest CT-scan (A) Showing mediastinal mass (yellow arrow), mass causing compression of left lower bronchus (black arrow), and (B) showing pericardial effusion (red arrow).

Further workup and management were done at a higher center with subspecialty services after transfer. Chest CT with contrast revealed a large anterior mediastinal mass with a mass extending into the paratracheal and subcarinal regions and involving the left hilum. It caused narrowing of the left main bronchus and collapse of the left lower lobe. There was a significant pericardial effusion. Figure 2 shows the chest CT findings. An echocardiogram confirmed a moderate to large pericardial effusion with early signs of cardiac tamponade, including right atrial collapse and marked respiratory variation in blood flow. Peripheral blood flow cytometry demonstrated T-cell acute lymphoblastic leukemia. Bone marrow aspiration and biopsy and cerebrospinal fluid analysis with flow cytometry confirmed the diagnosis of T-cell acute lymphoblastic leukemia with no central nervous system (CNS) involvement.

Due to the risk of airway or cardiac compromise, he was admitted to the pediatric intensive care unit for close monitoring. Intravenous fluids and allopurinol were started for tumor lysis prophylaxis, and a cefepime course was given for fever, given his functional neutropenia. A steroid course was given to reduce tumor burden. As cardiac tamponade progressed, a pericardial drain was placed for a few days. He was started on induction therapy as per the Acute Lymphoblastic Leukemia clinical trial protocol (AALL1231). He was given a transfusion of packed red blood cells during the hospital stay for severe anemia. Laboratory test trends showed resolution of hyperleukocytosis and improvement in hemoglobin levels post-transfusion, as shown in Table 2. The basic metabolic panel, uric acid, magnesium, calcium, and phosphorus trends were within the normal range and showed no evidence of tumor lysis syndrome. 

**Table 2:** Complete blood count after packed red blood cell transfusion and at the end of induction therapy TLC: total leucocyte count, ANC: absolute neutrophil count, RDW: red-cell distribution width.

Components	Reference range	Pre-transfusion	Post Transfusion	End of Induction phase
TLC	5.0-15.5 x 10³/µL	6.0	13.2	6.2
Neutrophil	22.0-69.0 %	63	38	71.0
Lymphocyte	16.0-67.0 %	31	39	17.1
Monocyte	4.0-12.0%	1	12	10.5
Eosinophil	0.0-4.0%	5	0	0.6
ANC	Above 1500/mm³	2520	5016	4402
Hemoglobin	10.2-13.7 g/dl	6.7	8.9	12.0
Red Blood cell count	3.90-5.00 x 10³/µL	2.28	2.85	3.87
Mean Corpuscular Volume	75.0-87.0 fL	90.4	97.2	88.6
Mean Corpuscular Hemoglobin Concentration	32.0-34.7 g/dL	32.5	32.1	35.0
Mean Corpuscular Hemoglobin	23.7-28.3 pg	29.4	31.2	31.0
RDW	12.5-14.9%	15.9	17.5	14.2
Platelet	150-400 x 10³/µL	94	239	350

**Figure 3:**
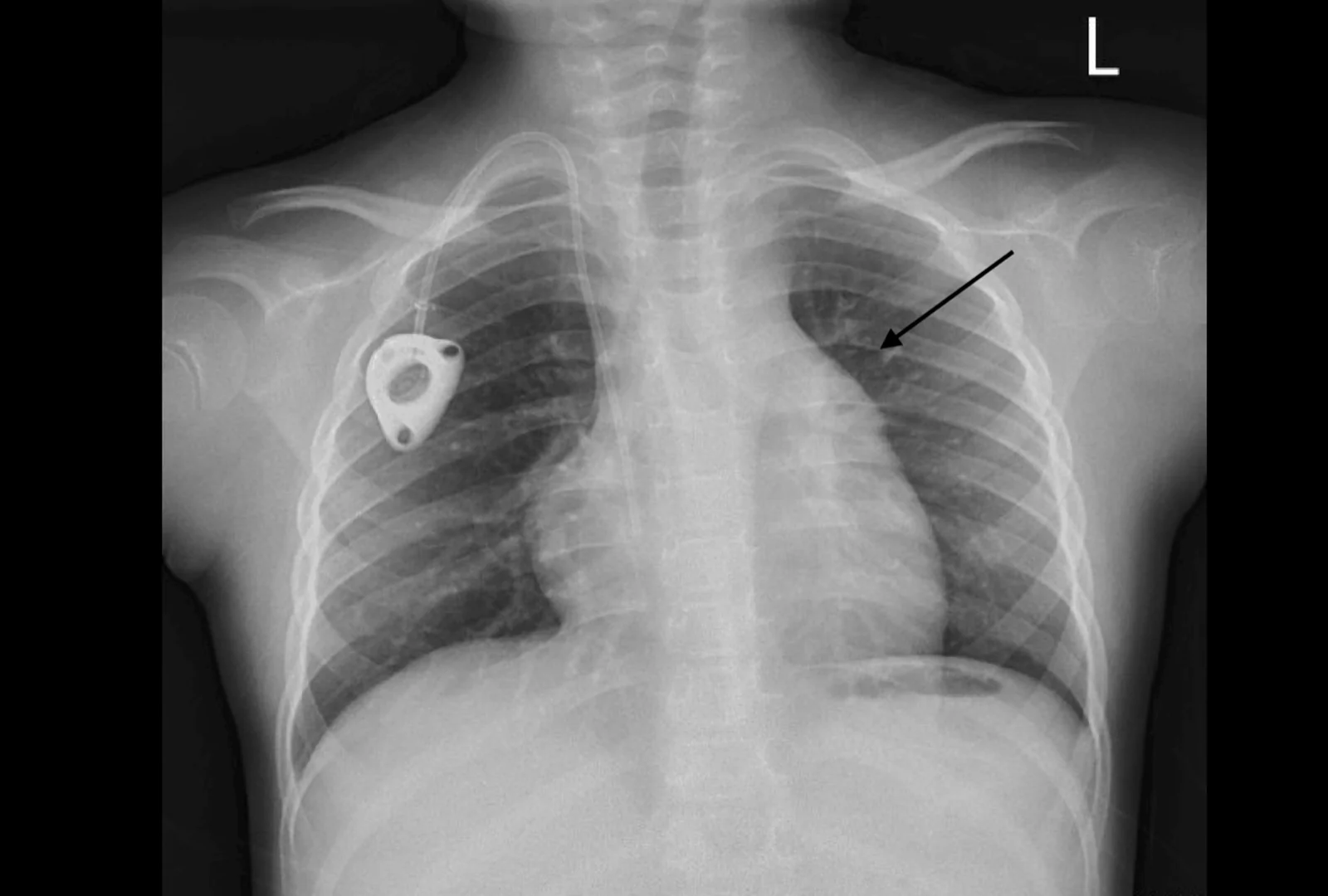
Frontal radiograph of chest Showing near-resolution of mediastinal mass. A mild bulge in the mediastinum, as shown by the black arrow, is present.

Following completion of induction therapy, a bone marrow study showed Minimal Residual Disease (MRD) negative status and negative cytology. A repeat chest x-ray showed near resolution of the mediastinal mass. The repeat chest x-ray is shown in Figure 3. 

## Discussion

This case highlights the importance of including acute leukemia as a part of the differential diagnosis in patients presenting with prolonged pneumonia-like symptoms. The patient presented initially with fever, cough, and chest pain. The chest radiograph was interpreted as pneumonia. However, the lack of clinical improvement after empiric antimicrobial treatment and the progressive widening of the mediastinum, lymphadenopathy, anemia, circulating blasts in the peripheral smear, and elevation in lactate dehydrogenase were more suggestive of an underlying hematologic malignancy than an infectious etiology [4].

Leukemic infiltration of the lung may cause cough, fever, hypoxemia, and radiographic infiltrates that are often indistinguishable from infection [3]. The phenomenon is more frequently described in the context of acute myeloid leukemia but can be seen in other leukemia subtypes too [6]. In the case of T-cell acute lymphoblastic leukemia (T-ALL) as well, an anterior mediastinal mass can cause airway compression, atelectasis, pleural effusion, or pericardial involvement that can mimic pneumonia clinically [2].

In pediatric acute lymphoblastic leukemia (ALL), it has been frequently observed to be associated with thoracic abnormalities. Smith et al. conducted a cohort study evaluating chest radiographs in children with newly diagnosed ALL and reported pulmonary opacities or infiltrates in 16.1%, pleural effusion in 11.1%, and mediastinal mass in 10.9% of children [4]. The study also reported that mediastinal mass, pleural effusion/thickening, and tracheal deviation/compression were more often associated with T-cell ALL than with B-cell ALL [4].

Clinicians should be aware of red flags that may indicate an alternative diagnosis other than uncomplicated pneumonia. These include failure to respond to adequate antimicrobial therapy, mediastinal widening, lymphadenopathy, hepatosplenomegaly, pallor, petechiae or ecchymosis, musculoskeletal pain, unexplained cytopenias, circulating blasts, and elevated lactate dehydrogenase [4]. In this case, persistent symptoms with cervical and axillary lymphadenopathy, anemia, marked predominance of blasts, and mediastinal widening were highly suspicious for leukemia [4].

In T-ALL, a large mediastinal mass requires careful attention to airway and hemodynamic risk. Pediatric patients may be clinically stable when awake and upright, but they are at risk for rapid deterioration with sedation, supine positioning, anesthesia, or tumor lysis [5]. Cross-sectional imaging and echocardiography are mandatory in the presence of mediastinal widening or cardiopulmonary symptoms [7]. In patients with pericardial effusion and tamponade, pericardiocentesis should be performed to allow definitive oncologic therapy [8]. Prophylaxis of tumor lysis syndrome should be started early. Fever in leukemia or neutropenia should be treated with empiric broad-spectrum antibiotics [9].

It is important to differentiate between leukemia mimicking pneumonia and infectious pneumonia as a complication in patients with known leukemia. Pulmonary infections are frequent during leukemia therapy and may be caused by bacterial, fungal, viral, or Pneumocystis jirovecii organisms and are influenced by immune status and phase of chemotherapy [8]. In the present case, the initial clinical picture was due to previously undiagnosed T-ALL with mediastinal and pericardial involvement rather than a confirmed infectious etiology.

## Conclusions

T-cell acute lymphoblastic leukemia can present initially as pneumonia, particularly when respiratory symptoms and radiographic abnormalities are the main features of presentation. If there is a failure to respond to antibiotics, mediastinal widening, lymphadenopathy, abnormal blood counts, circulating blasts, and elevated lactate dehydrogenase, there should be a high index of suspicion for leukemia, and urgent evaluation should be undertaken. Early recognition is very essential to prevent life-threatening complications, which include airway obstruction, cardiac tamponade, and tumor lysis syndrome.

## References

[1] Bradley JS, Byington CL, Shah SS (2011). The management of community-acquired pneumonia in infants and children older than 3 months of age: clinical practice guidelines by the Pediatric Infectious Diseases Society and the Infectious Diseases Society of America. Clin Infect Dis.

[2] Kovalski R, Hansen-Flaschen J, Lodato RF, Pietra GG (1990). Localized leukemic pulmonary infiltrates. Diagnosis by bronchoscopy and resolution with therapy. Chest.

[3] Aydin M, Flenaugh EL, Nichols M (2005). Hemoptysis, anemia and respiratory failure: a rare initial presentation of acute leukemia. J Natl Med Assoc.

[4] Smith WT, Shiao KT, Varotto E (2020). Evaluation of chest radiographs of children with newly diagnosed acute lymphoblastic leukemia. J Pediatr.

[5] Teachey DT, Pui CH (2019). Comparative features and outcomes between paediatric T-cell and B-cell acute lymphoblastic leukaemia. Lancet Oncol.

[6] Clarke RT, Van den Bruel A, Bankhead C, Mitchell CD, Phillips B, Thompson MJ (2016). Clinical presentation of childhood leukaemia: a systematic review and meta-analysis. Arch Dis Child.

[7] Inaba H, Teachey D, Annesley C (2025). Pediatric acute lymphoblastic leukemia, version 2.2025, NCCN Clinical Practice Guidelines in Oncology. J Natl Compr Canc Netw.

[8] Ben-Abraham R, Weinbroum AA, Augerten A (2001). Acute respiratory distress syndrome in children with malignancy--can we predict outcome?. J Crit Care.

[9] Baden LR, Swaminathan S, Almyroudis NG (2024). Prevention and treatment of cancer-related infections, version 3.2024, NCCN Clinical Practice Guidelines in Oncology. J Natl Compr Canc Netw.

